# The Application of CRISPR/Cas Systems for Antiviral Therapy

**DOI:** 10.3389/fgeed.2021.745559

**Published:** 2021-10-13

**Authors:** Helen J. E. Baddeley, Mark Isalan

**Affiliations:** Department of Life Sciences, Imperial College London, London, United Kingdom

**Keywords:** CRISPR, antiviral, Cas9, Cas13, HIV, hepatitis B, RNA virus, SARS-CoV-2

## Abstract

As CRISPR/Cas systems have been refined over time, there has been an effort to apply them to real world problems, such as developing sequence-targeted antiviral therapies. Viruses pose a major threat to humans and new tools are urgently needed to combat these rapidly mutating pathogens. Importantly, a variety of CRISPR systems have the potential to directly cleave DNA and RNA viral genomes, in a targeted and easily-adaptable manner, thus preventing or treating infections. This perspective article highlights recent studies using different Cas effectors against various RNA viruses causing acute infections in humans; a latent virus (HIV-1); a chronic virus (hepatitis B); and viruses infecting livestock and animal species of industrial importance. The outlook and remaining challenges are discussed, particularly in the context of tacking newly emerging viruses, such as SARS-CoV-2.

## Introduction

The current COVID-19 pandemic has reminded us of the power of emerging viruses and the need for new antiviral treatments. Ever since the development of CRISPR/Cas9 as an RNA-guided programmable genome editing tool (Jinek et al., 2012), there has been hope that it might be applied to directly cleave viral genomes (Doudna and Charpentier, 2014; Kennedy and Cullen, 2015). In nature, CRISPR/Cas systems are the adaptive immune systems within bacteria that protect against invading viruses and foreign nucleic acids (Horvath and Barrangou, 2010). Therefore, the logic follows that it might not be a step too far to exploit them to protect humans against viral infections.

**FIGURE 1:**
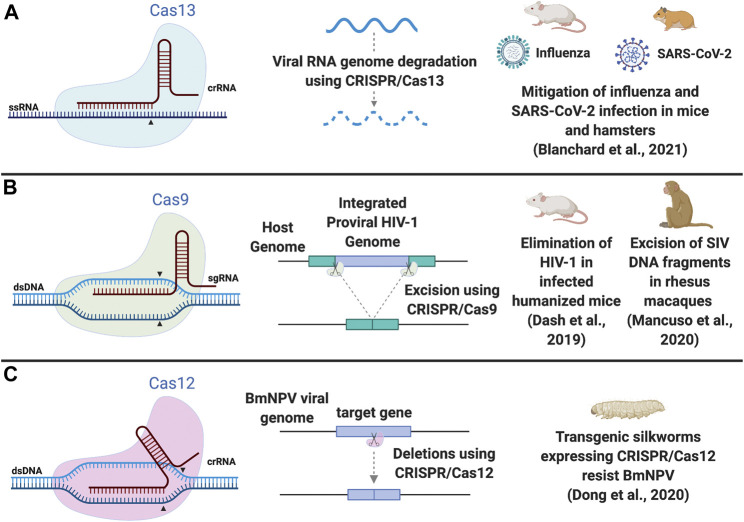
Schematic of CRISPR/Cas systems as antiviral therapy. **(A)** Cas13 cuts ssRNA and can be used to degrade viral RNA genomes. **(B)** Cas9 results in a blunt DNA cut and can excise integrated proviral HIV-1 DNA from the host genome, requiring 2 sgRNAs. Dual editing in the same cell may not always be efficient enough to excise the intervening segment of viral DNA but single site editing events, causing indels at the cut site, could inactivate viral replication. **(C)** Cas12 results in a staggered DNA cut and can delete regions in a target gene in the viral genome. Examples of *in vivo* studies in model animals are illustrated. Arrowheads indicate the cut structure of the effector protein. Abbreviations: dsDNA, doubled stranded DNA; ssRNA, single stranded RNA; sgRNA, single guide RNA; crRNA, CRISPR RNA; HIV-1, human immunodeficiency virus 1; SIV, simian immunodeficiency virus; BmNPV, Bombyx mori nucleopolyhedrovirus; SARS-CoV-2, severe acute respiratory syndrome coronavirus 2. Figure created with BioRender.com.

Within the diversity of natural CRISPR/Cas systems (Koonin et al., 2017), the Class II Cas endonucleases (single-protein effector) have recently been the subject of abundant research activity as possible antiviral therapeutics. The effectors Cas9 and Cas12 (both cut double stranded DNA, dsDNA) and Cas13 (cuts single stranded RNA, ssRNA) are suited against some DNA and RNA viruses, respectively (Figure 1; Table 1). The Cas13 subtypes vary in their PFS (protospacer-flanking sequence) requirements (Table 1). Of the Cas13 subtypes, Cas13d is the smallest which has implications for delivery (Yan et al., 2018). Since all Cas proteins are guided to their targets by an RNA molecule, which can easily be designed to bind conserved regions of viral genes, this makes them attractive scaffolds for flexible antiviral platforms.

**TABLE 1:** Properties of Class II CRISPR/Cas systems and summary of studies developing CRISPR/Cas antivirals. The PAM/PFS is critical for target recognition. The guide RNA is made up of the spacer (complementary to the target) and the scaffold (for binding to the Cas effector protein). *In vivo* studies are in bold. It is unclear whether any Cas proteins exist to cleave double-stranded RNAs, perhaps because of their complex secondary and tertiary structures; current antiviral Cas approaches must therefore focus on ssRNA targets within viruses, as shown here. Abbreviations: PAM, protospacer adjacent motif; PFS, protospacer-flanking sequence; BmNPV, Bombyx mori nucleopolyhedrovirus; HIV-1, human immunodeficiency virus-1; KSHV, Kaposi’s sarcoma-associated herpesvirus; PRRSV, porcine reproductive and respiratory syndrome virus; SARS-CoV-2, severe acute respiratory syndrome coronavirus 2.

*Target*	Type of CRISPR/Cas System	Effector Protein	Cut Structure	PAM/PFS	Guide Spacer Length/Total Guide Length (nt)	References (in alphabetical order, organised by virus)
dsDNA	II	Cas9	Blunt-ended	3′ GC-rich PAM	18–24/∼100	BmNPV: (**Chen et al., 2017**), (**Dong et al., 2018a**), (**Dong et al., 2018b**), (**Dong et al., 2019a**), (Dong et al., 2019b) Hepatitis B virus: (Kostyushev et al., 2019), (Kostyusheva et al., 2019), (**Li et al., 2018**), (**Liu et al., 2018**), (Schiwon et al., 2018), (**Stone et al., 2021**), (Suzuki et al., 2021), (**Yan et al., 2021**), (Yang et al., 2020)
						HIV-1: (**Bella et al., 2018**), (**Dash et al., 2019**), (Ebina et al., 2013), (Hu et al., 2014), (**Kaminski et al., 2016a**), (Kaminski et al., 2016b), (**Mancuso et al., 2020**)
						KSHV: (Liang et al., 2020)
dsDNA	V	Cas12	Staggered, 5′ overhang	5′ AT-rich PAM	18–25/42–44	BmNPV: (**Dong et al., 2020**) HIV-1: (Gao et al., 2020)
ssRNA	VI	Cas13	Cuts RNA	Cas13a: 3′ PFS: A, C or U. Cas13b: 5′ PFS: A, U or G; 3′ PFS: NAN or NNA (N = any base). Cas13d: no PFS requirement	22–30/52–66	Chikungunya virus: (Tng et al., 2020) Dengue virus: (Li et al., 2020), (Singsuksawat et al., 2021) HIV-1: (Yin et al., 2020) Hepatitis C: (Ashraf et al., 2021) Influenza A virus: (Abbott et al., 2020), (**Blanchard et al., 2021**), (Challagulla et al., 2021), (Freije et al., 2019), (Xu et al., 2021) Lymphocytic choriomeningitis: (Freije et al., 2019) PRRSV: (Cui et al., 2020) SARS-CoV-2: (Abbott et al., 2020), (**Blanchard et al., 2021**), (Wang et al., 2021), (Xu et al., 2021) Vesicular stomatitis virus: (Freije et al., 2019)

This article considers the different contexts in which CRISPR/Cas could be developed to directly target viruses, and the feasibility of each, as opposed to approaches that modify the host genome (for a review of those, see: (Chen et al., 2018)). Such novel strategies are sorely needed to fight the many serious viral infections that are of global public health concern. These infections often lack effective treatments or are showing resistance to existing antiviral drugs. Although this exciting field of research is still in its preclinical stages, it nonetheless holds great promise to defend us against viruses, one of which—SARS-CoV-2—is currently causing devastating effects worldwide (Hu et al., 2021).

## Main Text

### Examples of Acute Antiviral Therapy—RNA Viruses

RNA viruses have been an acute global priority since the rise of COVID and, in this context, Cas13 has drawn a lot of interest as an antiviral since it cuts ssRNA, such as that found in SARS-CoV-2 (Hu et al., 2021). In fact, ssRNA viruses make up the majority of viruses that can infect humans and so a Cas13 antiviral system was already developed and validated in mammalian cells even before COVID (Freije et al., 2019). For example, Cas13a and Cas13b were tested against 3 different pathogenic ssRNA viruses (lymphocytic choriomeningitis virus, influenza A virus and vesicular stomatitis virus) and CRISPR RNAs (crRNAs) targeting highly-conserved regions of the viral genome were found to be advantageous to avoid escape mutants. Moreover, multiplexing (targeting multiple loci), using pooled crRNAs, could reduce the chance of mutational escape and so the approach is highly adaptable to combat the possible evolution of viruses (Freije et al., 2019).

As soon as COVID emerged, work rapidly began to apply such approaches in this setting. The Cas13 antiviral strategy was first tested against SARS-CoV-2 in human lung epithelial cells, termed PAC-MAN (prophylactic antiviral CRISPR in human cells) (Abbott et al., 2020). This strategy uses pan-coronavirus crRNAs to guide Cas13d to degrade the viral genome and inhibit gene expression. This exploration occurred very early in the pandemic (April 2020) and had no access to live SARS-CoV-2. Hence, only synthesised fragments were used in this *in vitro* study. Since SARS-CoV-2 is a positive-sense virus, Cas13 could in principle be used to target both the genome and viral mRNAs to restrict viral replication. In order to target as many strains in the family as possible, Abbott and colleagues used bioinformatic screens to identify highly conserved regions across the viral genomes and found that just 6 crRNAs could target 91% of sequenced coronaviruses (Abbott et al., 2020).

Since then, numerous studies have used CRISPR to target SARS-CoV-2. In contrast to the pan-coronavirus approach, another group designed crRNAs guiding Cas13a in a highly specific, yet customizable, manner to the RNA of the SARS-CoV-2 spike protein (Wang et al., 2021). Similarly, the PAC-MAN approach used anti-SARS-CoV-2 Cas13d as a prophylactic, showing that it could be expressed in human lung epithelial cells *in vitro*, where it inhibited expression of SARS-CoV-2 fragments and live infection of influenza A virus (IAV; an RNA virus with a similar tropism as SARS-CoV-2) (Abbott et al., 2020). In a conceptually-different approach, mutant “deactivated” Cas13 (dCas13), lacking endonuclease activity, was shown to lower the expression of the spike protein without changing its RNA expression level, via perturbation of its translation (Wang et al., 2021).

Whilst these studies are exciting, the use of CRISPR/Cas systems against acute viruses remains ambitious and several challenges will need to be overcome to achieve functional antivirals *in vivo*. First, the issue of efficient delivery inside mammals is only beginning to be addressed. In 2021, Cas13a was demonstrated to mitigate influenza and SARS-CoV-2 infections *in vivo*, in rodent models (Blanchard et al., 2021) (Figure 1). Here, Cas13a was delivered as a synthetic mRNA, which is appealing since expression is transient and repeat dosing may be possible. Cas13a delivered after influenza infection was able to strongly knockdown viral RNA *in vivo*, showing for the first time the capability of Cas13a as a treatment post infection. However, in the SARS-CoV-2 *in vivo* experiments, Cas13a was delivered prior to infection and so was only tested as a prophylactic, rather than as a treatment. A second issue is that the immunogenicity of Cas proteins is not yet fully understood and more safety studies will be needed in order to establish how and when they may be safely expressed in humans (Mehta and Merkel, 2020). Third, as with most viruses, by the time symptoms begin to show after infection, and formal diagnosis confirms the identity of the virus, the viral load will likely already be very high and infection well-established, making elimination of the virus challenging. Having said that, momentum in the field is clearly building rapidly.

It should be noted that SARS-CoV-2 is not the only viral target of active study. For example, Cas13a has been used to successfully inhibit replication of hepatitis C virus (HCV) in human cells by targeting the highly conserved internal ribosomal entry site (Ashraf et al., 2021). CRISPR-based antivirals are promising here since HCV is challenging to treat due to its high genetic diversity. Another proof-of-concept study achieved inhibition of dengue virus replication by cleavage of the viral genome in mammalian cells (Li et al., 2020) and a similar approach was later carried out in human cells (Singsuksawat et al., 2021).

Yet another application of Cas13 is its use in insect vectors, such as *Aedes aegypti* and *Aedes albopictus* mosquitoes that transmit the RNA viruses chikungunya, dengue and Zika. In this context, Cas13b has been shown to knockdown chikungunya viral RNA in mosquito cells (Tng et al., 2020). This approach could potentially create virus-refractory mosquitoes. However, an unexpected result was that the guide RNAs alone could have an effect without Cas13b. Clearly, these works are still in progress, but the potential impact is great.

Finally, we note that RNA interference (RNAi) for silencing viral gene expression is a possible alternative therapy. RNAi, like Cas13, also offers a sequence-targeted antiviral approach in this case by binding to and degrading specific RNA transcripts (Levanova and Poranen, 2018). Short interfering RNA (siRNA) therapy was demonstrated to suppress symptoms and inhibit viral replication in a Rhesus macaque model of SARS-CoV (Li et al., 2005). Given that RNAi relies on native cellular machinery, it does not face the prospect of immunogenicity against the introduction of a foreign protein; this may be an issue for Cas13, particularly for repeated dosing. However, Cas13 seems to be favoured for clinical applications moving forward since it displays greater efficiency and specificity and lower off-target effects compared to RNAi (Abudayyeh et al., 2017; Konermann et al., 2018).

### DNA-Integrated Latent Virus Therapy—HIV

Because of some of the issues raised above with acute RNA viruses, the deployment of CRISPR/Cas as an antiviral may be better suited to targeting latent DNA viruses. HIV-1 is a good case study since significant preclinical progress is being made using CRISPR/Cas9 towards curing latent infection. Antiviral drugs do not by themselves eliminate the integrated DNA provirus from the host genome. Consequently, when antiretroviral therapy is stopped, viral rebound rapidly occurs and resistance can be a problem (Deeks et al., 2016). Therefore, CRISPR/Cas antivirals offer a much more flexible and nimble approach which may achieve eradication of the virus, potentially offering a permanent cure.

The original Cas9-based gene editing tool has the potential to excise or mutate latent dsDNA viruses (Figure 1). After successful excision of the integrated HIV-1 provirus in human myeloid cells (Ebina et al., 2013; Hu et al., 2014), suppression of viral replication was achieved in *ex vivo* cultured CD4^+^ T-cells from infected patients (Kaminski et al., 2016b), with suppression of viral gene expression in HIV-1 transgenic mice and rats (Kaminski et al., 2016a) and mice engrafted with infected peripheral blood mononuclear cells from HIV-1 positive patients (Bella et al., 2018). More recently, CRISPR/Cas9 has been combined with “long-acting slow-effective release antiviral therapy” (LASER ART) in humanized mouse models of HIV-1 infection (Dash et al., 2019). LASER ART consists of hydrophobic lipophilic antiretroviral prodrug nanoparticles that can reduce the dosing frequency of antiviral therapy from days to weeks, but it is not capable of eliminating HIV-1 by itself. The combination proved crucial to rid the mice of the virus and prevent rebound following cessation of antiviral therapy, since CRISPR/Cas9 alone was also not sufficient. In the first non-human primate study assessing this technology, adeno-associated viruses (AAVs) were used as delivery vectors. A single intravenous (IV) injection of AAV9-CRISPR/Cas9 was able to excise fragments of Simian Immunodeficiency Virus (SIV) integrated proviral DNA and the biodistribution was broad, reaching a range of tissues (Mancuso et al., 2020). Although a relatively small cohort was used, the results demonstrate real progress towards the goal of eliminating HIV-1 reservoirs in human patients to offer a permanent cure, and funding has been secured for phase 1/2 clinical trials.

Although Cas9 has been the main focus of anti-HIV-1 research, other Cas nucleases have recently been tested. An approach using Cas13a to degrade viral RNA was able to dramatically reduce the production of HIV-1 particles (Yin et al., 2020). Alternatively, Cas12a was shown to have better antiviral activity than Cas9 *in vitro* and caused mutations with distinct profiles (lack of pure insertions) (Gao et al., 2020). A limitation here is that the two Cas effectors have different PAM requirements so direct comparison is challenging. Overall, HIV therapy may be one of the first applications of CRISPR/Cas antivirals to actually reach human patients.

### Chronic Episomal Viral Therapy—Hepatitis B Virus

Another challenging virus that may be amenable to CRISPR/Cas approaches is Hepatitis B Virus (HBV). The HBV genome persists in hepatocytes in the form of covalently closed circular DNA (cccDNA), episomal DNA that is the replicative template, and it can also integrate its DNA into the host genome, where it is not replication competent but can still produce viral RNAs and proteins (Zhao et al., 2020). Therefore, this chronic infection represents a good testing ground for developing CRISPR/Cas antivirals.

The Cas9 ortholog must be carefully chosen when developing antiviral CRISPR/Cas therapies because it has been demonstrated to impact antiviral activity. For example, *Streptococcus thermophilus (St)* Cas9 was shown to be more effective at reducing HBV transcription and had no detectable off-target effects in contrast to *Streptococcus pyogenes (Sp)* Cas9 (Kostyushev et al., 2019). *St*Cas9 may be better suited for human therapeutics due to its lower mismatch tolerance and longer PAM requirement. Other factors can also impact antiviral activity, such as the impact of methylation of cccDNA on CRISPR/Cas9 activity against HBV; indeed, a recent study revealed an impairment of anti-HBV activity (Kostyushev et al., 2019).

Nonetheless, there are ongoing advances in such approaches. A study in liver-humanized mice using the smaller *Staphylococcus aureus (Sa)* Cas9, delivered by IV AAV, was able to improve survival of human hepatocytes, although reduction in HBV cccDNA did not reach statistical significance (Stone et al., 2021). This study used a more rigorous model of HBV infection, including the presence of cccDNA, compared to previous studies (Li et al., 2018; Liu et al., 2018). Thus, the successful targeting of HBV may be more challenging and shows that further optimisation is needed. For instance, small molecular inhibitors of DNA double strand break (DSB) repair pathways may prove useful as they can enhance CRISPR/Cas9 activity against HBV cccDNA (Kostyusheva et al., 2019).

Another consideration is that targeting integrated viral DNA, such as HBV and HIV, causes DSBs in the host genome which may lead to pathological chromosomal rearrangements (Kosicki et al., 2018). However, a recent development of the CRISPR/Cas system, base editing (Rees and Liu, 2018), can convert bases in specific loci without DSBs. This technology is therefore attractive for targeting integrated viruses since it could permanently silence gene expression by generating nonsense mutations, as demonstrated in both integrated HBV DNA and cccDNA (Yang et al., 2020). Moreover, to address safety concerns, the activity of CRISPR/Cas9 against HBV can be limited to the liver by using AAV8 which shows high liver tropism. Liver-specific promoters controlling the expression of CRISPR/Cas9 from AAV8 are effective as an additional method to restrict activity to the liver, as demonstrated in mice (Yan et al., 2021).

Practical applications of CRISPR/Cas9 against HBV will of course require efficient delivery. The recent development of high-capacity Adenoviral Vectors (HCAdVs) provides the opportunity for Cas9 and multiple gRNAs to be delivered in a single vector to combat HBV (Schiwon et al., 2018). Additionally, a lipid nanoparticle-based CRISPR/Cas ribonucleoprotein delivery nanoplatform has been developed, with potential for enabling large scale manufacturing (Suzuki et al., 2021). Here, the successful suppression of HBV DNA and cccDNA was demonstrated *in vitro*. The development of better delivery vehicles will be key to the roll-out of these technologies.

In addition to HBV, Cas9 has been shown to target Kaposi’s sarcoma-associated herpesvirus (KSHV/HHV8) episomal DNA *in vitro* (Liang et al., 2020). Specifically, sgRNAs against KSHV microRNA (miRNA) genes perturbed their function and resulted in the upregulation of host tumour-suppressor genes. This could be developed into a therapy for KSHV-associated malignancies, extending the applications for Cas9 to target episomal viral DNA.

### A More Ethically-Accessible Playground?—Antiviral Therapy for Animals

Substantial economic loss is endured because of viral infections in animals, leading to the development of some innovative strategies. Approaches directly targeting viruses, such as porcine reproductive and respiratory syndrome virus (PRRSV) (Cui et al., 2020), a major threat to the global pig industry, may be preferable in terms of regulatory approval, relative to human applications. In comparison to CRISPR/Cas approaches that target viruses topically, it is simpler to create germline-edited animals that either modify host genes required for viral infection (Burkard et al., 2017, 2018; Yang et al., 2018; Chen et al., 2019; Guo et al., 2019; Wang et al., 2019) or express CRISPR antivirals genomically. Thus, Cas13b is being investigated against PRRSV both as a method to cleave the viral RNA (Cui et al., 2020) and for a visual diagnostic method suitable for use in the field (Chang et al., 2020). Another example is the ability of stably expressed Cas13a to reduce IAV titres in chicken cells, using a combination of four crRNAs (Challagulla et al., 2021). This study suggests that the creation of germline transgenic chickens containing the CRISPR/Cas13a antiviral transgene might protect the poultry industry from highly pathogenic influenza strains.

The use of CRISPR/Cas as a prophylactic antiviral in humans is perhaps unlikely at this stage but it could offer tremendous protection in industrially-relevant animal species such as the silkworm (*Bombyx mori*). Transgenic silkworms have been created by integrating CRISPR/Cas9 into their genomes to target the Bombyx mori nucleopolyhedrovirus (BmNPV) genome. This provides resistance whilst retaining economically important characteristics for silk production (Chen et al., 2017; Dong et al., 2018a, 2019a). Interestingly, virus-inducible CRISPR/Cas9 systems have been developed against BmNPV to avoid the negative effects on larval and cocoon development that were seen with stable expression systems. Thus, the transgenic silkworm line contains Cas9 under the control of the baculovirus-inducible promoter 39 K, meaning that the system is activated only upon viral infection (Dong et al., 2018b). Other developments include the use of a multiplex CRISPR/Cas system to target several different silkworm viruses at the same time (Dong et al., 2019b). Additionally, although Cas12a has not yet been extensively applied to insect biotechnology, one Cas12a ortholog was shown to have improved antiviral activity against BmNPV, when compared to *Sp*Cas9 (Dong et al., 2020) (Figure 1).

Importantly, these studies are moving beyond testing the potential of CRISPR/Cas antiviral therapy in models of disease and into real natural viral infections. In summary, the development of CRISPR/Cas antivirals to treat animals is likely to develop more rapidly than for humans since transgenic animals can be generated so easily.

## Discussion

There has been a lot of promise surrounding applications of CRISPR/Cas, but the rapid progress on antivirals argues that these may genuinely be on a path to developing real-world applications.

As discussed in different viral contexts throughout this review, one of the major challenges which must be overcome to achieve effective treatments is the routine delivery of CRISPR/Cas components. A detailed discussion of this is outside the scope of this article but, as well as using standard viral delivery vectors (e.g. AAVs (Mancuso et al., 2020)), an innovative approach is the use of known receptor-ligand interactions to enable cell-type specific delivery (Rouet et al., 2018). This may allow specific targeting of infected cells by using the same receptor as for viral entry. Alternatively, albeit not in an antiviral context, is a study demonstrating successful base editing in primates after a single infusion of lipid nanoparticles (Musunuru et al., 2021). In addition to delivery challenges, pre-existing adaptive immunity to Cas9 in humans is clearly an important issue (Charlesworth et al., 2019). However, the use of transient “hit-and-run” delivery, combined with anti-inflammatories or targeted immunosuppression regimes may help to overcome these problems one day, as well as potentially applying alternative Cas proteins in patients.

Regarding the latter, there seems to be plenty left to be discovered. Earlier this year, two families of CRISPR/Cas13 effectors were discovered during metagenomic analysis of uncultivated microbes and one showed promising antiviral activity against SARS-CoV-2 and influenza (Xu et al., 2021). These effectors are much more compact which makes delivery easier. In addition, since the bacteria were located in hypersaline environments, humans are less likely to have pre-existing immunity to these orthologs compared to Cas9 and Cas12, which are from bacteria against which humans are commonly exposed.

Another discovery from scouring bacterial genomic and metagenomic sequences is Cas7-11, a programmable single-protein effector which resulted from the fusion of Cas7 with Cas11 (Özcan et al., 2021). Cas7-11 cuts RNA without showing any collateral activity or cell toxicity, in contrast to Cas13, and so is a promising new tool for combatting RNA viruses. Due to its large size, work will be required to shrink the enzyme for delivery.

In support of treating viral infections, rapid detection and diagnosis of viruses using CRISPR will likely achieve clinical impact more quickly (Gootenberg et al., 2018; Myhrvold et al., 2018). This approach is already being explored to rapidly detect SARS-CoV-2 with the ability to quantify viral load (Fozouni et al., 2021).

CRISPR/Cas antiviral platforms could offer tremendous improvement in flexibility against emerging viruses compared to vaccine development and screening for new antiviral drugs. Recently, tentative suggestions have been made that Ebola virus may unexpectedly exhibit latency (Fairhead et al., 2021), emphasising the need for an antiviral platform, such as CRISPR/Cas, to be poised for deployment. Whilst we have managed to develop vaccines in record time against COVID-19, CRISPR-based antiviral therapies may be important for tackling emerging viruses in the future. Since it may take years to reach clinical applications, we should begin to prepare now.

## Data Availability

The original contributions presented in the study are included in the article/Supplementary Material, further inquiries can be directed to the corresponding author.
